# Management of COVID-19-Positive Pediatric Patients Undergoing Minimally Invasive Surgical Procedures: Systematic Review and Recommendations of the Board of European Society of Pediatric Endoscopic Surgeons

**DOI:** 10.3389/fped.2020.00259

**Published:** 2020-05-07

**Authors:** Alessio Pini Prato, Andrea Conforti, Markus Almstrom, Wim Van Gemert, Maria G. Scuderi, Naziha Khen-Dunlop, Isabela Draghici, Mario Mendoza-Sagaon, Carlos Giné Prades, Fabio Chiarenza, Henri Steyaert

**Affiliations:** ^1^The Children Hospital, Azienda Ospedaliera SS Antonio e Biagio e Cesare Arrigo, Alessandria, Italy; ^2^Department of Medical and Surgical Neonatology, Ospedale Pediatrico Bambino Gesù, Rome, Italy; ^3^Department of Pediatric Surgery, Astrid Lindgren Children's Hospital, Karolinska University Hospital, Stockholm, Sweden; ^4^Department of Pediatric Surgery, University Medical Center Maastricht, Maastricht, Netherlands; ^5^Unit of Pediatric Surgery, Department GF Ingrassia, Hospital Policlinico, University of Catania, Catania, Italy; ^6^University Hospital Necker-Enfants Malades, Paris, France; ^7^Department of Pediatric Surgery, Maria Sklodowska Curie Hospital for Children, Bucharest, Romania; ^8^Department of Pediatric Surgery, Ospedale Regionale Bellinzona e Valli, Bellinzona, Switzerland; ^9^Digestive Pediatric Surgery, University Hospital Vall d'Hebron, Barcelona, Spain; ^10^Division of Pediatric Surgery, San Bortolo Hospital, Vicenza, Italy; ^11^Department of Pediatric Surgery, Queen Fabiola Children's Hospital, University of Brussels, Brussels, Belgium

**Keywords:** COVID 19, minimally invasive surgeries (MIS), pneumoperitoneum, safety, outbreak, pediatrics

## Abstract

**Introduction:** Hospital response to the COVID-19 outbreak has involved the cancellation of elective, deferrable surgeries throughout Europe in order to ensure capacity for emergent surgery and a selection of elective but non-deferrable surgeries. The purpose of this document is to propose technical strategies to assist the pediatric surgeons to minimize the potential aerosolization of viral particles in COVID-19 patients undergoing urgent or emergent surgical treatment using laparoscopic approaches, based on the currently available literature. The situation and recommendations are subject to change with emerging information.

**Materials and Methods:** The Scientific Committee and the Board of the European Society of Pediatric Endoscopic Surgeons gathered together in order to address the issue of minimally invasive surgery during this COVID-19 pandemic. A systematic search through PubMed, Embase, and World Wide Web of the terms “COVID-19,” “Coronavirus,” and “SARS-CoV-2” matched with “pneumoperitoneum,” “laparoscopy,” “thoracoscopy,” “retroperitoneoscopy,” and “surgery” was performed. Non-English language papers were excluded. A PRISMA report was performed. Criticalities were identified and a consensus was achieved over a number of key aspects.

**Results:** We identified 121 documents. A total of 11 full-text documents were assessed to address all concerns related to the adoption of minimally invasive surgery. All aspect of pediatric minimally invasive surgery, including elective surgery, urgent surgery, laparoscopy, thoracoscopy, retroperitoneoscopy, and pneumoperitoneum creation and maintainance were extensively addressed through systematic review. A consensus regarding urgent laparoscopic procedures, setting and operation techniques was obtained within the Committee and the Board.

**Conclusions:** The ESPES proposes the following recommendations in case minimally invasive surgery is needed in a COVID-19 positive pediatric patients: (1) consider conservative treatment whenever safely possible, (2) dedicate a theater, columns and reusable laparoscopic instrumentation to COVID-19 pediatric patients, (3) prefer disposable instrumentation and cables, (4) use low CO_2_ insufflation pressures, (5) use low power electrocautery, (6) prefer closed-systems CO_2_ insufflation and desufflation systems, and (7) avoid leaks through ports. These recommendations are subject to change with emerging information and might be amended in the near future.

## Background

The World Health Organization (WHO) recommendations to contain the coronavirus (COVID-19) outbreak include social distancing, avoidance of crowding and staying at home. Reducing the flow of people within countries also increases the likelihood of successful infection control. Restrictions implemented in Italy on the 7th of March 2020 included limitations on all personal activities. Similar limitations were implemented by other countries during the following weeks.

Most hospitals have been involved in treatment of COVID-19 patients. In areas where the virus outbreak has been particularly severe, hospital infrastructure has been acutely remodeled to enable the direction of resources toward the care of COVID-19 patients. The need to contain the outbreak, treat cases and protect healthcare personnel have featured as prime considerations.

In spite of this scenario, ordinary illnesses, trauma, and diseases requiring surgery continue to prevail alongside COVID-19. For reasons that are not yet understood, children appear to be less susceptible and generally the course of the disease is less severe (1).

Hospital response to the outbreak has involved the cancellation of elective, deferrable surgeries throughout Europe in order to ensure capacity for emergent surgery and a selection of elective but non-deferrable surgeries. This has been regarded as essential to avoid the complications of time-dependent diseases in terms of survival and functional results. These have included certain pediatric liver conditions, malignancies, congenital malformations, and functional disorders amongst other acute problems that cannot be postponed for a reasonable amount of time to pass safely through the pandemic.

Patients suffering from these diseases will continue to be treated according to the COVID-19 infection prevention strategies of their local institutions. In procedures involving laparoscopy and thoracoscopy, the possibility of viral contamination through aerosolization during carbon dioxide (CO_2_) insufflation and electrocautery has been raised as a concern that is being closely reviewed worldwide.

The purpose of this document was to propose technical strategies to assist the pediatric surgeons to minimize the potential aerosolization of viral particles in COVID-19 patients undergoing urgent or emergent surgical treatment using laparoscopic approaches, based on the currently available literature. The situation and recommendations are subject to change with emerging information.

## Methodology

### Systematic Literature Review

PubMed and Embase were searched for the terms “COVID-19,” “Coronavirus,” and “SARS-CoV-2” matched with “pneumoperitoneum,” “laparoscopy,” “thoracoscopy,” “retroperitoneoscopy,” and “surgery” with all possible combinations. Non-English language papers and documents were excluded.

### World Wide Web Search

Guidelines, recommendations or statements published by scientific societies, including adult societies and their websites were searched using a combination of the same terms as above.

### Preferred Reporting Items for Systematic Reviews and Meta-Analyses (PRISMA)

This schematic methodology was used to report significant and homogeneous results of the systematic review.

### Identification of Criticalities

The papers and documents extracted from the web were assessed with regard to indications on elective surgery, on urgent and emergent surgery using laparoscopy, thoracoscopy, and retroperitoneoscopy as well as on the risk of aerosolization during CO_2_ insufflation with regard to these specific techniques.

### Proposed Statement

Pediatric surgeons belonging to the Scientific Committee of the European Society of Pediatric Endoscopic Surgeons proposed a summary of the evidence and technical strategies for laparoscopic procedures to assist pediatric surgeons during this pandemic.

## Results

### PRISMA

**Figure 1:**
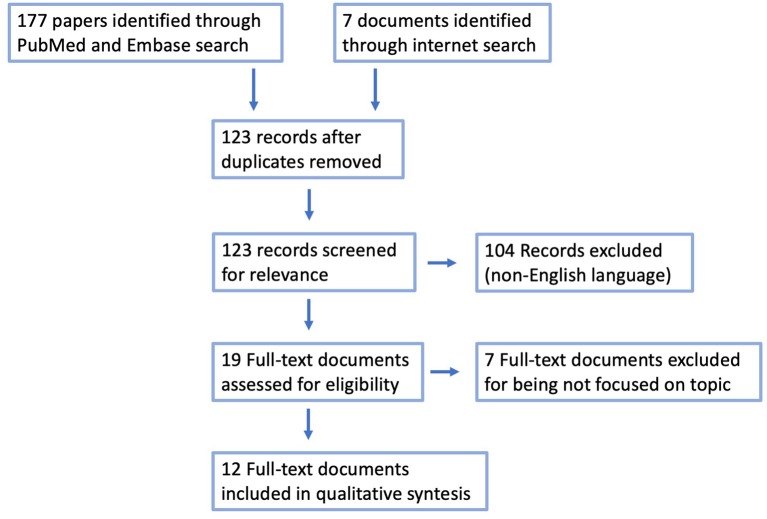
PRISMA module for systematic review.

One-hundred and 16 papers were identified through PubMed and 61 through Embase searches. A further seven documents regarding reccomendations or position statements from Scientific Societies worldwide were identified with a web search. After duplicate removal, a total of 123 papers were screened for relevance. A total of 12 full-text documents [8 peer-reviewed papers (2–9) and 5 documents (10–14)] were assessed and turned out to be eligible to address all concerns related to the adoption of minimally invasive surgery (involving pneumoperitoneum, prenumoretroperitoneum, or pneumothorax creation) in pediatric patients (Table 1). See Figure 1 for PRISMA details.

**Table 1:** List of papers included in systematic review (rows) and areas of assessment (columns).

**References**	**Age**	**Elective surgery**	**Urgent surgery**	**Laparoscopy**	**CO_**2**_ insufflation**	**Other aspects**
Spinelli and Pellino (2)	Adults	Only oncologic surgery	Indications for surgery unchanged	Might be safer	Better than open provided closed system is used	NA
Repici et al. (3)	Adults	NA	NA	NA	NA	Guidelines for endoscopy
Pellino and Spinelli (4)	Adults	Warns delays over 90 days	NA	NA	NA	NA
Iacobucci (5)	Adults	Suspended for 3 months since 15th of April 2020	NA	NA	NA	NA
Wang and Du (6)	NA	NA	NA	NA	Highest risk if aerosol spreading is confirmed	NA
Zheng et al. (7)	Adults	Only oncologic surgery	Emergencies and cancer surgery	Allowed	Careful aerosolization in closed cavity with eletrocauthery	NA
Lee et al. (8)	NA	NA	NA	NA	NA	Consider swab test for patients and parents/visitors
Wen and Li (9)	NA	Canceled	Emergencies with specific considerations	NA	NA	Recommend negative pressure theaters, anesthesia with paralysis and avoid preoxygenation
BAPES (10)	Children	NA	NA	Feasible with care	Be careful on aerosolized CO_2_	NA
American College of Surgeons section of Pediatric Surgery (11)	Children	Postponed	List of urgent and semi-urgent procedures	NA	NA	NA
American College of Surgeons (12)	Adults	Postponed	List of deferrable urgent procedures	NA	NA	
Society of American Gastrointestinal and Endoscopic Surgeons (13)	Adults	Postponed to the end of peak pandemic	Emergencies and cancer surgery	Allowed	Keep CO_2_ insufflation pressure low	NA

### Addressed Criticalities and Issues

#### Elective Surgery

This aspect was addressed by 5 papers (2, 4, 5, 7, 9) and 3 documents (11–13) that underlined that all elective procedures should be postponed. Iacobucci in particular, stated that UK stopped all elective surgery of at least 3 months since 15th of April 2020 thus postponing all elective procedures in the second half of July 2020 (5). However, in certain circumstances, including malignancy, it was identified that delaying surgery would negatively influence the outcome and these therefore represented urgent surgical patients (4). The only document addressing time-dependent diseases in pediatric population is that from the American College of Surgeons that divided diseases according to emergencies, semi-urgent diseases and elective deferrable diseases (11).

#### Urgent Surgery

This aspect was addressed by 3 papers (2, 7, 9) and 3 documents (11–13) that agreed that urgent surgery should be performed. Given the high risk of surgery (both for the patients and for the medical personnel) in case of COVID-19-positive patients, the American College of Surgeons has recommended that conservative management should be followed when it is safely possible (12). As stated above, the American College of Surgeons (section of Pediatric Surgery) also identified pediatric diseases requiring urgent, semi-urgent or elective surgery on an empirical basis (11). Local institutional infection control policies for surgical theaters in confirmed or suspected COVID-19 patients should be followed.

#### Laparoscopy

This aspect was addressed in detail by only 2 papers (2, 7) and 2 documents (10, 13) and therefore the evidence regarding laparoscopy was very limited and of low quality. None contraindicated the use of laparoscopy in COVID-19-positive patients, but all underlined a risk of aerosolization of viral particles during CO_2_ insufflation. This aspect was supported by a recent paper by Li and colleagues who reported a higher concentration of 0.3 and 0.5 μm particles in laparoscopy compared to laparotomy after 10 min of electrosurgical treatment (14). All papers supported the use of closed systems (i.e., Stryker Pneumoclear Smoke Evacuation High Flow Tube® sets or ConMed Airseal® delivery system) and filters for CO_2_ evacuation. Zheng and co-workers suggested low pressure CO_2_ insufflation, low-power electrocautery use, minimize Trendelenburg and avoid leakage from trocar sites as much as possible (7). Finally, the use of intracorporeal anastomoses has been advocated by some in order to minimize the risk of dangerous fecal aerosolization (2). Particular concern is raised in case of combined laparoscopic/endoscopic procedures as those required in case of symptomatic choledocholithiasis requiring urgent surgery (3). Of note, Spinelli and colleagues suggested that open surgery could be even worse in terms of contamination when diathermy, electrosurgery and ultrasonic sealing device are used (2).

#### Thoracoscopy and Retroperitoneoscopy

None of the papers focused on this aspect in details.

#### CO_2_ Insufflation

This aspect was addressed by 3 papers (2, 6, 7) and 2 documents (10, 13). All Authors suggested that the risk of aerosolization was palpable. In particular, a paper by Wang and colleagues, based on a peculiar transmission modality reported in Mongolia, suggested that COVID-19 may transmit through aerosol directly making prevention and control much more difficult (6). Based on this incertitude, all supported the use of the highest degree of dedicated and adequate Personal Protection Equipment (PPE) in order to contain the risk of contamination, according to local institutional infection control policies for surgical theaters in confirmed or suspected COVID-19 patients.

### ESPES Consensus on Laparoscopic Procedures in Children During COVID-19 Outbreak

#### Indications (Level of Agreement—High)

Whenever a COVID-19-positive patient can have their surgery safely delayed until the patient has fully recovered from the infection, this should be preferred. In acute surgical conditions, if a conservative management (i.e., uncomplicated appendicitis) is safely possible and does not negate the outcome for the patient, this should be considered. Ideally, patients should be tested for COVID-19 before procedures, if this is practically feasible.

#### Setting (Level of Agreement—High)

Dedicated theater facilities (including laparoscopic column camera and cables) for suspected or confirmed COVID-19-positive patients should be arranged, if possible. Appropriate filtering and ventilation are important, and negative pressure rooms may also be useful. Unnecessary night-time operating should be avoided in order to decrease the workload of anesthesiologists and nurses likely to be needed to reinforce adult teams.

#### Procedure and Operation Technique (Level of Agreement—High)

In case of thoracoscopy, retroperitoneoscopy, or laparoscopy we recommend, whenever possible:

- Dedicate to COVID-19-positive patients a laparoscopic column (see above)
- Maintain low pressure CO_2_ and/or use of suspension techniques
- Keep power settings of electrocautery as low as possible
- Use disposable insufflation cables with high-capacity filters
- Use customized or commercially available closed CO_2_ evacuation systems with filters
- Avoid leaks through trocar
- Avoid the use of trocars without valves
- Favor the use of disposable trocars, best if with sealing balloons
- Prefer staplers instead of loops (appendectomy) to minimize the risk of leaks through the ports
- Use disposable trocars in the context of a COVID-19-positive patient, if available. If reusable trocars are used, they should be cleaned separately from other surgical equipment
- Aspiration of the residual CO_2_ at the end of the operation in a closed system with filters.

*Strict use of PPE and infection prevention strategies* as for conventional open surgery and anesthesia for aerosol producing maneuvers should be followed. It is strongly recommended to follow the guidelines for droplet and airborne precautions of local institutions. This is particularly true for airway and endoscopic procedures. For specific detailed guidelines we recommend WHO as well as local institutional guidelines.

## Discussion

In order to contain COVID-19 outbreak and to support the requirements of various European Health Systems in order to deal with this dramatic pandemic, it is necessary to adhere to a number of indications that apply to pediatric surgery and have been addressed by most governments.

1. Postpone all elective surgery according to institutional or local guidelines
2. Consider a number of time-dependent diseases requiring elective non-deferrable surgery
3. Prepare to encounter COVID-19-positive patients requiring urgent surgical treatment.

On the ground of these considerations, it is likely that a number of COVID-19-positive pediatric patients will require some sort of urgent surgical treatment. For this aspect, institutions will need to set procedures and protocols in order to be prepared to cope with all critical issues. Of note, some papers addressed the issue of viral spreading during CO_2_ pneumoperitoneum creation during laparoscopy in case of Hepatitis (15) or Human Immunodeficiency Viruses (16), both in adults and children. They found laparoscopy to be safe, even safer than conventional laparotomy. Nonetheless, as those viruses are not transmitted by droplets nor aerosolization (they are not respiratory viruses), the translation of these considerations to COVID-19 setting is over-reaching and should be avoided. As time being there is lack of strong scientific evidences on which to support one approach over the other. As a matter of facts, we can provide recommendations based on reasonable hypothesis, common sense, and shared feelings. Based on these limitations, we strongly suggest Institutions and Scientific Societies keep monitoring emerging evidence.

The choice of approach, whether laparoscopic or open should be based on careful consideration of the balance between the benefits and risks specific to the procedure, and the risk of viral contamination/aerosolization to the staff. The proven benefits of minimally invasive surgery in certain procedures (i.e., reduced length of stay and complications) should be considered, as well as the capacity for ultrafiltration of any aerosolized particles that may also pose a similar or greater risk if an open approach is adopted (2, 3, 6, 7, 9).

## Conclusions

As a summary, as pediatric surgeons belonging to the European Society of Pediatric Endoscopic Surgeons (ESPES) we suggest the following concerning laparoscopic, thoracoscopic, and retroperitoneoscopic procedures in COVID-19 positive patients:

- Dedicate a theater to perform urgent procedures, avoid unnecessary night-time operating
- Consider conservative treatments, whenever safely possible
- Dedicate minimally invasive columns and reusable instrumentation
- Prefer disposable instrumentation and cables
- Use low-pressure CO_2_ insufflation
- Use low-power electrocautery
- Prefer closed-systems CO_2_ insufflation and desufflation systems
- Avoid leaks through ports (valved and/or balloon ports, small skin incisions).

The aims of this document are to provide optimal patient care, maintain safety for all perioperative staff, and, as much as possible, help contain the COVID-19 pandemic. The situation and recommendations are subject to change with emerging information and might be amended in the near future.

## Data Availability Statement

The original contributions presented in the study are included in the article/supplementary materials, further inquiries can be directed to the corresponding author/s.

## Author Contributions

AP performed the Systematic Review. AP, AC, MA, and WV drafted the paper. MS, NK-D, ID, MM-S, CG, FC, and HS reviewed the draft and contributed to the consensus. All authors reviewed and approved the final version of the manuscript.

## Conflict of Interest

The authors declare that the research was conducted in the absence of any commercial or financial relationships that could be construed as a potential conflict of interest.
